# Dietary Patterns Are Differentially Associated with Atypical and Melancholic Subtypes of Depression

**DOI:** 10.3390/nu13030768

**Published:** 2021-02-26

**Authors:** Aurélie M. Lasserre, Marie-Pierre F. Strippoli, Pedro Marques-Vidal, Lana J. Williams, Felice N. Jacka, Caroline L. Vandeleur, Peter Vollenweider, Martin Preisig

**Affiliations:** 1 Department of Psychiatry, Lausanne University Hospital and University of Lausanne, 1011 Lausanne, Switzerland; Marie-Pierre.Strippoli@chuv.ch (M.-P.F.S.); Caroline.Vandeleur@chuv.ch (C.L.V.); martin.preisig@chuv.ch (M.P.); 2 Department of Medicine, Internal Medicine, Lausanne University Hospital and University of Lausanne, 1011 Lausanne, Switzerland; Pedro-Manuel.Marques-Vidal@chuv.ch (P.M.-V.); Peter.Vollenweider@chuv.ch (P.V.); 3 Barwon Health, IMPACT—The Institute for Mental and Physical Health and Clinical Translation, Deakin University, Geelong 3220, Australia; l.williams@deakin.edu.au (L.J.W.); f.jacka@deakin.edu.au (F.N.J.)

**Keywords:** major depressive disorder, depression subtypes, atypical, melancholic, dietary patterns, population-based study, cross-sectional, cardiovascular risk factors

## Abstract

Diet has been associated with the risk of depression, whereas different subtypes of depression have been linked with different cardiovascular risk factors (CVRFs). In this study, our aims were to (1) identify dietary patterns with exploratory factor analysis, (2) assess cross-sectional associations between dietary patterns and depression subtypes, and (3) examine the potentially mediating effect of dietary patterns in the associations between CVRFs and depression subtypes. In the first follow-up of the population-based CoLaus|PsyCoLaus study (2009–2013, 3554 participants, 45.6% men, mean age 57.5 years), a food frequency questionnaire assessed dietary intake and a semi-structured interview allowed to characterize major depressive disorder into current or remitted atypical, melancholic, and unspecified subtypes. Three dietary patterns were identified: Western, Mediterranean, and Sweet-Dairy. Western diet was positively associated with current atypical depression, but negatively associated with current and remitted melancholic depression. Sweet-Dairy was positively associated with current melancholic depression. However, these dietary patterns did not mediate the associations between CVRFs and depression subtypes. Hence, although we could show that people with different subtypes of depression make different choices regarding their diet, it is unlikely that these differential dietary choices account for the well-established associations between depression subtypes and CVRFs.

## 1. Introduction

Depression is a common mental disorder affecting more than 250 million people worldwide [1]. In addition to the suffering related to this disorder and the significant consequences on quality of life, depression is associated with high rates of cardiovascular diseases [2,3] and mortality [4,5] making it a major public health issue. Depression has a multi-causal pathogenesis including genetic, environmental and lifestyle factors and much remains to be done in understanding contributing factors and physiopathology [6]. A promising approach is to consider the heterogeneity of depression in terms of symptom manifestations, course and response to pharmacological treatment [7,8,9]. Indeed, depression subtypes are likely to be differentially associated with biological mechanisms. The atypical subtype is more strongly related to cardio-metabolic (e.g., the metabolic syndrome) and to inflammation dysregulations, the melancholic subtype to a dysfunction of the hypothalamic-pituitary-adrenal (HPA) axis [7,10,11,12,13,14,15].

Evidence has accumulated to suggest that diet plays a role in the onset of depression. Cross-sectional and prospective studies have revealed consistent results, with healthy diets, characterized by a high consumption of vegetables, fruit, fish, and whole grains, being associated with less depressive symptoms and a lower risk of developing depressive symptoms [16,17,18]. In contrast, a dietary pattern characterized by a high consumption of red or processed meat, refined grains, sweets, high-fat dairy products, butter, potatoes, and high-fat gravy, a low intake of fruit and vegetables, frequently referred to as “Western”, is likely to be associated with an increased risk of depression [19]. A randomized prevention trial on cardiovascular diseases including 7447 people aged 55 to 80 years partly confirmed these results by showing that a Mediterranean diet supplemented with nuts could exert a beneficial effect on the risk of depression in patients with type 2 diabetes, but not in the entire sample [20].

A frequently reported limitation of studies on the association between diet and depression is the high level of heterogeneity in the assessment of diet quality and depression as well as in the selection of study samples [21,22]. A recent meta-analysis of observational studies conducted by Nicolaou et al. addressed the most frequent limitations by standardizing variables in the individual studies and including a wide array of potential confounders/covariates in the analyses [18]. The authors documented inverse associations between three, a priori defined, dietary patterns, using scores or indices measuring healthy diet, and depressive symptoms in cross-sectional as well as prospective analyses over a timeframe of 5–6 years [18]. However, this meta-analysis was based on studies assessing depressive symptoms through rating scales rather than diagnostic interviews to elicit formal criteria for major depressive episodes according to the Diagnostic and Statistical Manual of Mental Disorders, fourth edition (DSM-IV) [23]. Most existing studies investigating the association between dietary patterns and depression used scales, which do not generally allow for characterization into depression subtypes and do not consider the frequent occurrence of comorbid mental disorders. To assess diet habits, a wide spectrum of methods has been used. Hence, finding the most robust and reproducible method to capture such a subtle equilibrium remains a challenge. Dietary patterns may be more informative for investigating diet-disease relationships if they reflect the synergistic and correlated effects of separate nutrients and foods [24]. Indeed, patterns with the same labels can be defined by different food components or by different amounts of the same components.

Many studies using different methods have consistently found two dietary patterns: The ‘Prudent’ or ‘Mediterranean’ pattern, characterized by a high intake of vegetables, fruit, legumes, fish and seafood, and whole grains, and a less-healthy pattern, frequently referred to as a ‘Western’ pattern, characterized by a high intake of red meat, processed meat, butter, potatoes, refined grains, and high-fat dairy products. [25,26,27]. The ‘Mediterranean’ diet, compared with the ‘Western’ pattern has been associated with more favorable biological profiles, slower progression of atherosclerosis, and reduced incidence of cardiovascular diseases; ultimately the “Western” diet, could also increase mortality [25,27,28,29].

Considering that a healthy diet is known to lower the risk of cardiovascular diseases [29] as well as the risk of depression [16], and given that the melancholic and the atypical subtypes of depression are associated with different cardio-metabolic risk profiles [13,15], an examination of the association between dietary patterns and subtypes of depression is crucial to ultimately understand their interplay with different cardiovascular risk factors (CVRFs). Therefore, using data from a large population-based study, the aims of the present study were to (1) identify dietary patterns with exploratory factor analysis, (2) assess the cross-sectional associations between these identified dietary patterns and depression subtypes, and (3) examine the potentially mediating effect of dietary patterns in the cross-sectional associations between CVFRs and depression subtypes.

## 2. Materials and Methods

### 2.1. Participants

We used data from the first follow-up visit of CoLaus|PsyCoLaus [30,31], which took place between 2009 and 2013, as the assessment of dietary intake was first introduced at this time point. This prospective cohort study was designed to investigate mental disorders and cardiovascular diseases in the community and to determine their associations. The baseline sample was randomly selected from the residents of the city of Lausanne (Switzerland) aged between 35 and 75 years in 2003 according to the civil registry. This assessment consisted of a physical followed by a psychiatric evaluation. Out of the 5064 participants who accepted the physical evaluation, 4580 (90%; mean age 58 years, 54% women) also completed the food frequency questionnaire (FFQ) and could be included in the factor analysis to identify dietary patterns. Among them, 3554 (78%) also took part in the psychiatric evaluation and thus were included in the analyses investigating the association between depression subtypes and dietary patterns. In comparison to the participants included in the physical follow-up, those who were included in these analyses were statistically significantly younger (57.5 vs. 58.6 years) and less physically inactive (68.2% vs. 72.6%).

### 2.2. Physical Evaluation

The physical evaluation, conducted by trained nurses, included a standardized interview, a physical exam, as well as the completion of questionnaires. During the interview, information on socio-demographic characteristics, current medication, alcohol consumption (number of alcoholic drinks per week), smoking status (current or former smoking) and physical activity was elicited. Socio-economic status (SES) was determined using the Hollingshead scale [32]. This scale considers the level of education and employment status of the participant and of his or her spouse. Caucasian origin was defined as having both parents and grandparents born in a restricted list of countries (available from the authors). Participants were considered physically inactive if they reported physical activity for less than 20 min twice a week. Body weight and height were measured with participants barefoot and in light indoor clothes. Body weight was measured in kilograms to the nearest 100 g using a Seca^®^ scale (Hamburg, Germany). Height was measured to the nearest 5 mm using a Seca^®^ height gauge (Hamburg, Germany). Blood pressure (BP) was measured using an Omron^®^ HEM-907 automated oscillometric sphygmomanometer after at least a 10 min rest in a seated position, and the average of the last two measurements was used. Venous blood samples were drawn after an overnight fast to measure the levels of glucose, HDL-cholesterol, LDL-cholesterol, triglycerides, and insulin. The diagnosis of obesity was assigned according to WHO diagnostic criteria, i.e., a body-mass index of 30 kg/m^2^ or more. The diagnosis of hypertension was assigned according to the Joint National Committee (JNC) criteria, i.e., a systolic BP of 140 or above, a diastolic BP of 90 mmHg or above, or any antihypertensive treatment [33]. The diagnosis of diabetes was assigned according to the Expert Committee on the Diagnosis and Classification of Diabetes criteria [34], i.e., a fasting glucose level of 7 mmol/L or above or a antidiabetic treatment. The diagnoses of dyslipidemia was assigned according to the National Cholesterol Education Program (NCEP) Expert Panel (Adult Treatment Panel III, ATP III) criteria, i.e., fasting blood triglycerides of 2.2 mmol/L or above, LDL-cholesterol of 4.1 mmol/L or above, HDL-cholesterol of 1 mmol/L or less, or any hypolipidemic treatment [35]. Dietary intake was assessed using a self-administered, semi-quantitative FFQ which also included portion sizes [36]. This FFQ was validated in the Geneva population [36,37]. Briefly, this FFQ assesses the dietary intake of the previous four weeks and consists of 97 different food items which account for more than 90% of the intake of calories, proteins, fat, carbohydrates, alcohol, cholesterol, vitamin D and retinol, and 85% of fibers, carotene, and iron. For each item, consumption frequencies ranging from “less than once during the last 4 weeks” to “2 or more times per day” were provided, and participants also indicated the average serving size (smaller, equal or bigger) compared to a reference size. Each participant was asked to complete the FFQ prior to their appointment, which was checked for completion on the day of the study visit.

### 2.3. Psychiatric Evaluation

Interviewers were master-level psychologists, who were trained over a two-month period. They collected diagnostic information on mental disorders using the French version of the semi-structured Diagnostic Interview for Genetic Studies (DIGS) [38,39]. The DIGS was completed with the anxiety disorder sections of the Schedule for Affective Disorders and Schizophrenia—Lifetime Version (SADS-L) [40]. Psychiatric diagnoses were assigned according to the DSM-IV. Atypical features are defined by mood reactivity and two of the following four symptoms: weight gain or increase in appetite, hypersomnia, leaden paralysis, and interpersonal rejection sensitivity. The melancholic features are defined by a loss of pleasure or lack of reactivity and three of the following symptoms: a distinct quality of mood (despair), depression worse in the morning, early-morning awakening, psychomotor agitation, or retardation, weight loss, and guilt. We could not consider the criterion “distinct quality of depressed mood” because it was not assessed in the DIGS. A major depressive disorder (MDD) was considered to be current if a major depressive episode was present at the time of the dietary assessment, and remitted otherwise. Participants were divided into seven mutually exclusive groups according to the course (current, remitted, never) and the clinical features of a lifetime MDD: (1) current atypical, (2) current melancholic, (3) current unspecified (not meeting criteria for atypical or melancholic features), (4) remitted atypical (at least one atypical episode without any melancholic episode), (5) remitted melancholic (at least one melancholic episode without any atypical episode), (6) remitted unspecified (at least one unspecified episode without any melancholic or atypical episode or at least one melancholic and one atypical episode), (7) no MDD.

### 2.4. Ethical Approval

The institutional Ethics Committee of the University of Lausanne approved the baseline and follow-up CoLaus|PsyColaus studies (reference 16/03, decisions of 13 January and 10 February 2003 and reference 33/09, decision of 23 February 2009, respectively). The study was performed in agreement with the Helsinki declaration and its former amendments, and in accordance with the applicable Swiss legislation. All participants gave their signed informed consent before participating in the study.

### 2.5. Statistical Analysis

Food items endorsed by less than 10% of the participants were excluded from the factor analysis. For the identification of dietary patterns, the principal factor method was used, followed by a promax (oblique) rotation (Factor procedure in the Statistical Analysis System, version 9.4; SAS Institute, Inc., Cary, NC, USA). A scree test was used to determine the number of factors to retain. Food items with a loading of at least 0.3 on a given factor were retained in the promax rotation. Univariate analyses to compare characteristics across depression subtypes relied on chi-square tests and ANOVA for dichotomous or continuous variables, respectively. Multinomial logistic regression models were used to assess the associations between the MDD subtypes, as the dependent variable (reference group: never MDD), and the dietary patterns scores, as independent variables. The first model was adjusted for socio-demographic characteristics (age, sex, ethnicity, SES and living alone) and the second model was further adjusted for lifestyle factors (physical inactivity, smoking, and alcohol consumption). To assess the potentially mediating effect of these dietary patterns in the associations between CVRFs (obesity, diabetes, hypertension and dyslipidemia) and MDD subtypes, the effect sizes (ORs) of these associations were compared between two multinomial logistic regression models: the first model was adjusted for socio-demographic characteristics (age, sex, ethnicity, SES and living alone) and lifestyle factors (physical inactivity, smoking, and alcohol consumption), the second model was further adjusted for dietary patterns.

## 3. Results

### 3.1. Factor Analysis of FFQ

The factor analysis was performed with the data of the 4580 participants (54% women, mean age of 57.7 years), who completed the 97 items FFQ at the physical follow-up. Four items (garlic pills, bran supplement, vitamin E supplement and fat-free milk) were consumed by less than 10% of participants and were thus excluded from the analysis. The scree plot and the interpretability of the factors suggested a solution with 3 meaningful factors (Table 1). The first two factors include 18 and the third factor 10 items. The first factor, labeled Western, included fried potatoes, sausages, processed and red meats, refined-grain cereals, pre-prepared meals, beer, and sugar-sweetened beverages, whereas the second factor, labeled Mediterranean included fruit and vegetables, fish, seafood, olive oil and poultry, the third factor, labeled Sweet-Dairy, was mainly made up of butter, high- and low-fat dairy products, jam and honey, chocolates, cookies, pastry, and cakes. The three factors explained 31%, 21% and 11% of the variance of food intake, and their standardized Cronbach alpha coefficients were 0.76, 0.76, and 0.67, respectively.

### 3.2. Association between Dietary Patterns and MDD Subtypes

Table 2 describes the sample of 3554 participants included in the analyses between dietary patterns and depression subtypes. Among the 1666 participants with a lifetime MDD, 16% met criteria for the atypical, 28% for the melancholic and 56% for the unspecified subtypes. At the time of the assessment, 15.0% of those who met lifetime criteria for MDD were experiencing a current episode. Diagnostic groups differed significantly according to several socio-demographic (sex, age, living alone), lifestyle (smoking and alcohol consumption) characteristics.

Table 3 reveals that regardless of the adjustments, increasing Western dietary scores were associated with a decreased likelihood of experiencing a current melancholic episode or having met criteria for remitted melancholic MDD. In contrast, in the fully adjusted model (Model 2), increasing Western dietary scores were associated with an elevated likelihood of experiencing a current atypical episode but not with the reporting of remitted atypical MDD. In addition, increasing Sweet-Dairy dietary scores were positively associated with an elevated likelihood of experiencing a current melancholic episode but not with the reporting of remitted melancholic MDD. No association was found between Mediterranean dietary score patterns and depression status in either model.

### 3.3. Potentially Mediating Effect of Dietary Patterns in the Associations between CVRFs and MDD Subtypes

The associations between the four CVRFs obesity, diabetes, hypertension, and dyslipidemia and MDD subtypes before and after adjustment for dietary patterns are presented in Table 4. In the first model, obesity was associated with a higher likelihood of having a remitted atypical MDD, whereas diabetes was associated with a decreased likelihood of experiencing a current unspecified depressive episode. Adding the dietary pattern scores into the model (Model 2) did not affect these significant associations. However, a new significant association appeared between diabetes and an increased likelihood of experiencing a current melancholic episode.

## 4. Discussion

In a large population-based sample, we identified three dietary patterns, Western, Mediterranean, and Sweet-Dairy, which were differentially associated with subtypes of MDD. Participants with an atypical depression tended to eat unhealthier food (Western) during depressive episodes but not after the offset of these episodes, whereas those with a melancholic depression did not only tend to avoid unhealthy (Western) food during depressive episodes but also after these episodes. Moreover, the latter group of depressive participants tended to eat more uncooked, picnic-style food (Sweet-Dairy) during depressive episodes. However, we did not find evidence for a mediating effect of these dietary patterns in the associations between CVRFs and MDD subtypes. To our knowledge, this is the first population-based study to assess dietary patterns and their relationship with depression subtypes as well as the potentially mediating effect of these patterns in the associations between CVRFs and MDD subtypes. By showing that atypical and melancholic depression subtypes do not only differ on their cardio-metabolic profiles [13], but also on a lifestyle characteristic as fundamental as diet, our results extend the understanding to the differentiated profiles hypothesis of the atypical and melancholic subtypes [15].

The dietary patterns identified by factor analysis in the present study were consistent with those previously reported: a Western pattern characterized by preferred consumption of red meat, processed meat, butter, potatoes, refined grains, and high-fat dairy products and a Mediterranean, sometimes named Prudent pattern, with preferred consumption of vegetables, fruit, legumes, fish, and whole grains [25,26,27,41,42]. The Sweet-Dairy dietary pattern, based mainly on uncooked food, has also been frequently documented. A similar pattern, called “bread and cookies” was found to be the first factor in a population-based cohort in the Netherlands based on more than 100,000 adults [43]. Our finding of inverse associations of the Western pattern with atypical and melancholic subtypes is in line with the results of the BiDirect Study, the only previous study which assessed links between depression subtypes and diet quality, assessed using an 18-item FFQ. Indeed, among 840 patients with depression and 820 controls from this clinical study [44], patients with the melancholic depression subtype had a higher diet quality than those with the atypical subtype of depression. However, neither of the two groups of depressive patients significantly differed from the non-depressed controls [44].

Splitting depression into current and remitted revealed two different types of associations between depression subtypes and dietary patterns in our study. The association between the atypical subtype and Western diet as well as the association between the melancholic subtype and Sweet-Dairy diet were only present among participants exhibiting a current depressive episode, but not among those with remitted depression. This suggests a temporary phenomenon with a change of dietary patterns restricted to the timespan of depressive episodes. Given that the Western diet, which is has been shown to be a risk factor for cardio-metabolic diseases [25], was not observed after the offset of depressive episodes with atypical features, which generally have a limited duration, it is unlikely that this food pattern can significantly contribute to the well-known association between atypical MDD and the subsequent development of cardio-metabolic diseases.

In contrast, the association between melancholic MDD and low intake of food according to the Western dietary pattern was not only present during but also after the remission of melancholic episodes. This is a relevant finding given that a long-lasting dietary pattern is more likely to have an impact on health than a temporary change that only occurs during depressive episodes. However, the cross-sectional nature of our data does not allow us to determine the direction of this association. Indeed, the lower intake of food according to the Western dietary pattern could already have preceded the onset of melancholic MDD and might have even been involved in its development. Conversely, it could have been a consequence of melancholic episodes. However, given that previous prospective studies suggested that the Mediterranean diet is a preventive factor and the Western diet a risk factor for depression [16,17,18,19,20], it seems unlikely that a low intake of food according to the Western dietary pattern is a risk factor for melancholic MDD. The reverse direction of association appears more likely, meaning that people with melancholic MDD may durably reduce their intake of food according to the Western dietary pattern. However, the temporal sequence of the association between melancholic MDD and Western diet first needs to be established in prospective studies. In contrast to melancholic MDD, the positive association between atypical MDD and Western diet was limited to the duration of depressive episodes. This association could be attributable to factors associated with both dietary patterns and this depression subtype. For instance, the FTO gene, which has been shown to be associated with obesity and atypical depression, might also have an impact on dietary choices during depressive episodes [42]. Similarly, environmental or psychological factors could predispose to both the choice of diet and vulnerability to depression.

Previous studies have provided evidence for cross-sectional [15,45,46] and longitudinal associations [13,47,48] between cardio-metabolic diseases and particularly the atypical MDD subtype. Hence, the observed cross-sectional association between remitted atypical MDD and the risk of obesity is in line with these previous results. In contrast, the association between current melancholic MDD and an elevated risk of diabetes as well as the negative association between current unspecified MDD and diabetes have not been previously reported. Our analysis revealed that it is unlikely that these associations between MDD subtypes and CVRFs were mediated by dietary patterns. This is not surprising given that three out of four established associations between MDD subtypes and dietary patterns were limited to the duration of depressive episodes and it is unlikely that changes of dietary patterns restricted to such relatively brief periods represent serious risk factors for cardio-metabolic diseases. Only the Western dietary pattern was associated with the remitted MDD subtype, i.e., the negative association between this subtype and remitted melancholic MDD. Although it is plausible to hypothesize that a lower long-term intake of Western diet in people with melancholic MDD may contribute to the lower risk of obesity in these people as compared to those with atypical MDD [13,15,48], our data do not provide support to this hypothesis. Indeed, adding the effects of dietary patterns into the model did not influence the size of the association between any CVRF and remitted melancholic MDD. Therefore, these findings advocate for other hypotheses in understanding the differences of cardio-metabolic profiles among different MDD subtypes, such as an unmeasured environmental factor, or the demonstrated genetic variability among the atypical and melancholic subtypes [49].

Our study has several limitations. First, as already mentioned, the cross-sectional setting of the study hinders conclusions regarding the direction of these associations. Second, participants and non-participants differed in age and degree of physical activity, which may have biased the measured associations, although we have adjusted our analyses for these variables.

## 5. Conclusions

People with the atypical or melancholic depression do not only have different cardio-metabolic profiles [13,15], but they also differ in their dietary patterns. However, the dietary changes associated with MDD subtypes were essentially limited to the timespan of the depressive episodes and did not mediate the associations between MDD subtypes and CVRFs. Hence, although we could show that people with different subtypes of depression make different choices regarding their diet, it is unlikely that these differential dietary choices account for the well-established associations between depression subtypes and CVRFs.

## Figures and Tables

**Table 1:** Selected explanatory factor loadings for the 3-factor model (after promax rotation, *n* = 4580).

	Western	Mediterranean	Sweet-Dairy
Fries	0.559	−0.065	−0.070
Sausage	0.458	−0.012	0.092
Dried sausage, salami	0.453	−0.083	0.142
Burger	0.421	0.079	0.004
Pizza	0.405	0.052	0.060
White bread	0.402	−0.156	0.062
Beer	0.381	−0.113	−0.096
Steaks	0.368	0.110	−0.066
Pâté, terrine	0.367	−0.017	0.097
Croissant, pastry	0.366	−0.064	0.077
Ham, stew	0.365	0.076	0.076
Roast chicken	0.361	0.033	−0.038
Fried fish	0.356	0.076	−0.019
Sweetened beverages	0.339	−0.093	0.079
Ravioli	0.331	0.101	0.120
Mayonnaise (as condiment)	0.325	−0.063	0.197
Cervelas (sausage)	0.324	−0.081	−0.025
Pasta	0.316	0.117	0.122
Carrots	−0.014	0.485	0.059
Green beans, spinach	0.128	0.468	−0.145
Lean fish	−0.039	0.457	−0.032
Cauliflower, broccoli	0.023	0.448	−0.009
Tomatoes	0.151	0.433	−0.216
Green salad	0.043	0.404	−0.017
Salmon (smoked or fresh)	0.020	0.392	−0.003
Kiwi	−0.177	0.389	0.058
Berries	0.078	0.363	−0.083
Olive oil	−0.013	0.347	0.046
Shrimps, sea food	0.187	0.346	−0.153
Peach, nectarine, apricot, melon	0.113	0.345	−0.175
Chicken breast	0.011	0.344	−0.061
Banana, apple, pear, plum, grapes	−0.168	0.338	0.196
Wheat semolina, couscous	0.023	0.331	0.113
Peas, corn	0.282	0.324	−0.050
Avocado	0.030	0.322	0.052
Dressing (as condiment)	0.037	0.314	0.044
Butter (as condiment)	0.123	−0.077	0.509
Jam, honey	−0.069	0.075	0.482
Chocolate	−0.003	0.028	0.435
Cookies	0.079	0.050	0.388
Heavy cream (as condiment)	0.207	−0.005	0.372
Fruit galette	0.162	0.116	0.341
Cheese	0.122	−0.043	0.336
Tea, infusion	−0.242	0.221	0.321
Biscuit, cake	0.174	0.044	0.317
Butter (for cooking)	0.247	−0.075	0.316

**Table 2:** Characteristics of the participants who responded to the food frequency questionnaire and the psychiatric interview (*n* = 3554).

		Major Depressive Disorder Subtypes								
	All	Current Atypical	Current Melancholic	Current Unspecified	Remitte Atypical	Remitted Melancholic	Remitted Unspecified	Never Depressed	χ^2^/F	*p*-Value
	(*n* = 3554)	(*n* = 53)	(*n* = 56)	(*n* = 125)	(*n* = 196)	(*n* = 380)	(*n* = 746)	(*n* = 1998)		
Male, %	45.6	24.5	30.4	32.0	26.5	34.7	37.3	54.4	χ^2^_6_ = 154.2	<0.001
Age (y), mean (SD)	57.5 (10.4)	55.9 (9.0)	54.2 (9.8)	54.7 (9.3)	54.1 (9.5)	55.9 (9.6)	56.1 (9.7)	59.0 (10.8)	F_6_ = 17.1	<0.001
Non-Caucasians, %	6.6	9.4	12.5	4.8	6.6	7.4	8.0	5.7	χ^2^_6_ = 10.0	n.s.
SES, mean (SD) ^a^	3.5 (1.2)	3.3 (1.1)	3.2 (1.4)	3.4 (1.3)	3.6 (1.1)	3.6 (1.2)	3.6 (1.1)	3.5 (1.2)	F_6_ = 1.9	n.s.
Living alone, %	27.5	28.3	44.6	40.8	29.1	30.3	30.7	24.3	χ^2^_6_ = 35.3	<0.001
Physical inactivity, % ^b^	68.2	66.0	67.9	70.4	70.4	67.4	66.2	68.7	χ^2^_6_ = 2.5	n.s.
Smoking status, %										
Current	20.6	30.2	32.1	24.0	16.8	22.9	23.9	18.6	χ^2^_6_ = 21.2	0.002
Former	38.2	34.0	37.5	37.6	41.8	36.3	38.3	38.4	χ^2^_6_ = 2.1	n.s.
Number of alcohol drinks per week, mean (SD)	6.4 (8.1)	3.5 (4.7)	5.2 (7.1)	5.4 (6.8)	4.9 (5.2)	5.9 (7.7)	5.5 (6.5)	7.1 (9.0)	F_6_ = 7.0	<0.001

Key: y: years, SES: socio-economic status, SD: standard deviation, n.s.: not significant. Χ_2_/F: chi-square tests (dichotomous variables)/ANOVA (continuous variables). ^a^ A value of 3 represents an SES of III (middle class) on the Hollingshead scale. ^b^ Less than 20 min twice a week.

**Table 3:** Associations between dietary patterns and subtypes of major depressive disorder (*n* = 3554).

	Major Depressive Disorder Subtypes						
	Current Atypical OR (95CI)	Current Melancholic OR (95CI)	Current Unspecified OR (95CI)	Remitted Atypical OR (95CI)	Remitted Melancholic OR (95CI)	Remitted Unspecified OR (95CI)	Never Depressed
Model 1							
Western	1.35 (0.99, 1.83)	0.65 * (0.47, 0.91)	1.06 (0.85, 1.31)	1.01 (0.85, 1.21)	0.87 * (0.76, 1.00)	0.96 (0.87, 1.06)	1 (ref.)
Mediterranean	1.09 (0.82, 1.45)	0.81 (0.61, 1.07)	0.93 (0.76, 1.12)	1.01 (0.86, 1.19)	1.00 (0.89, 1.12)	1.00 (0.91, 1.09)	1 (ref.)
Sweet-Dairy	0.96 (0.71, 1.29)	1.33 (0.99, 1.79)	0.97 (0.79, 1.18)	0.95 (0.81, 1.13)	1.02 (0.90, 1.15)	0.99 (0.90, 1.08)	1 (ref.)
Model 2							
Western	1.44 * (1.05, 1.96)	0.64 ** (0.45, 0.90)	1.07 (0.86, 1.34)	1.05 (0.87, 1.26)	0.87 * (0.76, 1.00)	0.98 (0.88, 1.09)	1 (ref.)
Mediterranean	1.09 (0.82, 1.45)	0.83 (0.62, 1.10)	0.93 (0.76, 1.13)	1.00 (0.85, 1.18)	1.01 (0.89, 1.13)	1.00 (0.91, 1.10)	1 (ref.)
Sweet-Dairy	0.97 (0.72, 1.32)	1.39 * (1.03, 1.88)	0.97 (0.79, 1.19)	0.94 (0.80, 1.11)	1.03 (0.91, 1.17)	0.99 (0.90, 1.09)	1 (ref.)

Key: OR = odds ratio, 95CI = 95% confidence interval. * *p* < 0.05, ** *p* < 0.01. Model 1: adjusted for socio-demographic factors (age, sex, ethnicity, socio-economic status, living alone). Model 2: adjustments of model 1 + for behavioral factors (physical inactivity, smoking status, alcohol consumption).

**Table 4:** Association between cardiovascular risk factors and subtypes of major depressive disorder (*n* = 3554).

	Major Depressive Disorder Subtypes						
	Current Atypical	Current Melancholic	Current Unspecified	Remitted Atypical	Remitted Melancholic	Remitted Unspecified	Never Depressed
	OR (95CI)	OR (95CI)	OR (95CI)	OR (95CI)	OR (95CI)	OR (95CI)	
Model 1							
Obesity	1.55 (0.76, 3.12)	0.87 (0.40, 1.91)	1.17 (0.70, 1.96)	1.77 ** (1.20, 2.61)	1.11 (0.80, 1.53)	0.98 (0.76, 1.27)	1 (ref.)
Diabetes	2.11 (0.89, 5.01)	2.28 (0.96, 5.39)	0.27 * (0.08, 0.90)	1.12 (0.62, 2.04)	0.81 (0.51, 1.29)	1.18 (0.86, 1.62)	1 (ref.)
Hypertension	0.93 (0.48, 1.83)	1.50 (0.80, 2.81)	1.18 (0.77, 1.81)	0.95 (0.66, 1.37)	0.91 (0.70, 1.19)	0.86 (0.70, 1.06)	1 (ref.)
Dyslipidemia	1.37 (0.75, 2.49)	0.85 (0.47, 1.54)	1.24 (0.84, 1.83)	1.06 (0.76, 1.47)	1.01 (0.79, 1.28)	1.09 (0.91, 1.32)	1 (ref.)
Model 2							
Obesity	1.47 (0.72, 3.02)	0.92 (0.41, 2.06)	1.14 (0.68, 1.92)	1.76 ** (1.19, 2.60)	1.13 (0.81, 1.57)	0.98 (0.76, 1.27)	1 (ref.)
Diabetes	2.06 (0.86, 4.93)	2.61 * (1.09, 6.29)	0.28 * (0.08, 0.90)	1.12 (0.62, 2.03)	0.82 (0.52, 1.31)	1.18 (0.86, 1.62)	1 (ref.)
Hypertension	0.92 (0.46, 1.81)	1.64 (0.87, 3.09)	1.17 (0.76, 1.80)	0.95 (0.66, 1.37)	0.92 (0.71, 1.20)	0.86 (0.70, 1.06)	1 (ref.)
Dyslipidemia	1.35 (0.74, 2.47)	0.90 (0.49, 1.64)	1.23 (0.83, 1.83)	1.06 (0.76, 1.47)	1.02 (0.80, 1.30)	1.10 (0.91, 1.32)	1 (ref.)
“Western”	1.36 (0.99, 1.87)	0.62 ** (0.44, 0.87)	1.07 (0.85, 1.34)	1.01 (0.84, 1.22)	0.87 * (0.75, 1.00)	0.98 (0.88, 1.09)	1 (ref.)
“Mediterranean”	1.08 (0.81, 1.43)	0.81 (0.61, 1.08)	0.94 (0.77, 1.14)	1.01 (0.86, 1.19)	1.01 (0.89, 1.14)	0.99 (0.91, 1.09)	1 (ref.)
“Sweet-Dairy”	1.03 (0.75, 1.42)	1.48 * (1.09, 2.02)	0.97 (0.79, 1.20)	0.98 (0.83, 1.16)	1.03 (0.91, 1.17)	0.99 (0.90, 1.10)	1 (ref.)

Key: OR = odds ratio, 95CI = 95% confidence interval. * *p* < 0.05, ** *p* < 0.01. Model 1: adjusted for socio-demographic factors (age, sex, ethnicity, socio-economic status, living alone) and behavioral factors (physical inactivity, smoking status, alcohol consumption). Model 2: adjustments of model 1 + for the 3 dietary patterns.

## Data Availability

Due to the sensitivity of the data and the lack of consent for online posting, individual data cannot be made accessible. Only metadata can be made available in digital repositories. However, individual data requests for metadata can be performed via the study website www.colaus-psycolaus.ch.
